# Primary unifocal thymic Rosai-Dorfman disease: an extremely rare challenge in diagnostic practice

**DOI:** 10.1186/s13019-023-02381-4

**Published:** 2023-10-10

**Authors:** Qian Liu, Fengxiang Liao, Yong Liu, Yang Cheng, Chubo Qi

**Affiliations:** 1 Department of Pathology, Jiangxi Provincial People’s Hospital, the First Affiliated Hospital of Nanchang Medical College, No. 152, Aiguo Road, Nanchang, 330006 China; 2 Department of Nuclear medicine, Jiangxi Provincial People’s Hospital, the First Affiliated Hospital of Nanchang Medical College, Nanchang, 330006 China

**Keywords:** Rosai-Dorfman disease (RDD), Thymus, Pathologic diagnosis

## Abstract

Rosai-Dorfman disease (RDD) is currently considered a group of neoplastic diseases of unknown etiology, with monoclonal proliferation of histiocytes, showing unique histopathologic features and varying clinical presentation. Primary thymic RDD is an extremely rare extranodal form of this disorder. In this study, we describe the case of an otherwise healthy 64-year-old Chinese man who presented with an isolated, asymptomatic soft tissue density lesion in the anterior mediastinum detected by computed tomography. Histology of the surgical specimen revealed infiltration of thymic tissue by sheets of large histiocytes with mixed lymphocytes and plasma cells, and background fibrosis. Immunohistochemical staining of the histiocytes was positive for S100, CD68, CD163, OCT2 and cyclin D1, but negative for CD1a and BrafV600E expression, thus supporting a diagnosis of RDD. Primary thymic RDD is extremely rare and may be a diagnostic challenge when presenting as mediastinal lesion.

## Introduction

Rosai-Dorfman disease (RDD) was first described by Pierre Paul Louis Lucien Destombes in 1965 as a disorder involving “adenitis with lipid excess.” In 1969, Juan Rosai and Ronald Dorfman recognized this condition as a distinctive disorder of histiocytic proliferation [1]. RDD is a rare, non-Langerhans cell histiocytic disorder of unknown etiology. A portion of RDD demonstrates monoclonal histiocytic proliferation, associated with gene mutations in the mitogen-activated protein kinase (MAPK) signaling pathway. RDD is most frequently diagnosed in children and adolescents who present with massive bilateral cervical lymphadenopathy, often with associated fever, mild anemia, elevated sedimentation rate and polyclonal gammopathy. The histopathology of RDD lesions is characterized by tissue infiltration with large histiocytes and a mixed inflammatory background. The histiocytes show abundant pale cytoplasm with emperipolesis, and express S100, CD68, CD163, OCT2 and cyclin D1. Of note, RDD-associated histiocytes are more strongly positive for CD163 than CD68 [2, 3]. Although classic RDD was originally described as a form of lymphadenopathy, more than one-quarter of patients diagnosed with this disorder present with extranodal manifestations [2]. The most frequent sites of extranodal involvement are the skin, upper respiratory tract, orbits, testes and bones [4]. In this report, we present a rare case of primary RDD detected in the thymus.

## Case presentation

**Fig. 1:**
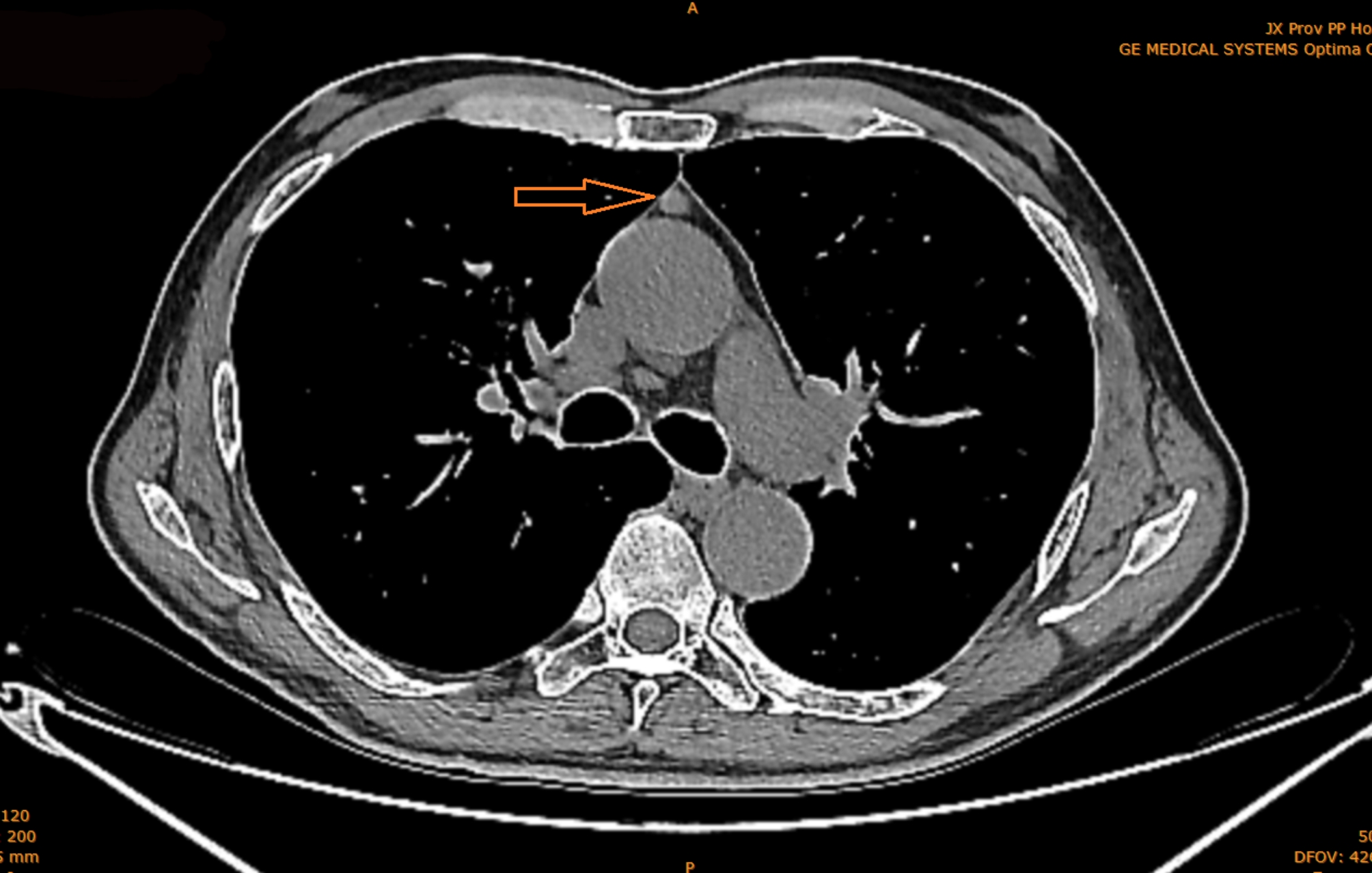
Computed tomography scan, showing an isolated and well circumscribed soft tissue density (1.2 × 1.0 cm) in the anterior mediastinum

A 64-year-old man presented to our hospital for a routine health check-up. A unifocal soft tissue density in the thymus, measuring 1.2 cm × 1.0 cm, was detected as an incidental lesion by whole-body computed tomography (Fig. 1). The patient reported that he had smoked one pack of cigarettes per day for more than 30 years. Although he coughed occasionally, he denied hoarseness, chest pain or dyspnea. A physical examination and all laboratory test findings were within normal limits. A diagnosis of thymoma was suspected clinically, and the patient underwent surgery in our hospital.

**Fig. 2:**
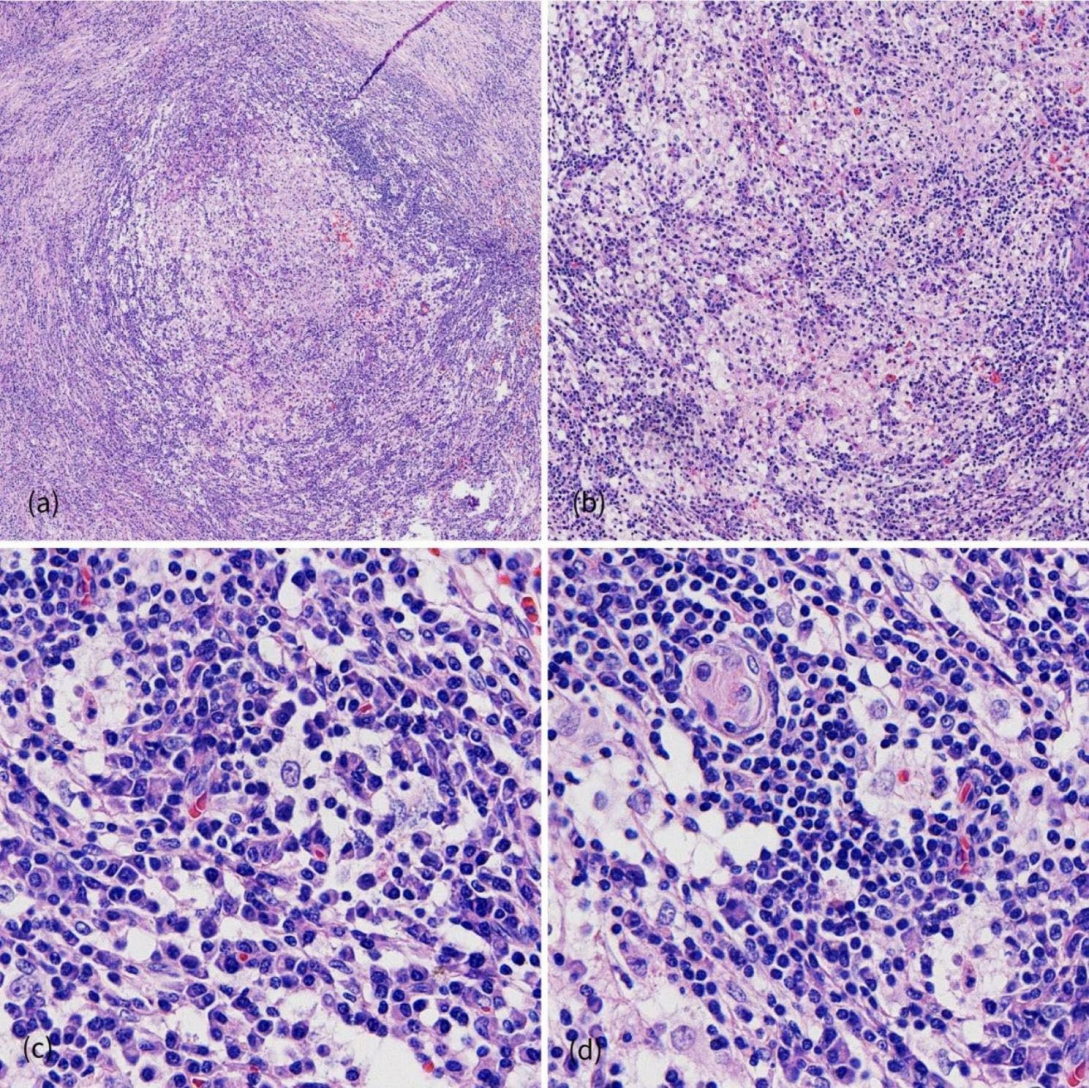
Morphological features of primary thymic RDD. (**a, b**) Histological section, showing clusters of large histiocytes accompanied by lymphocytes and plasma cells with a background of fibrosis (4×, 10×). (**c, d**) The histiocytes exhibited large round nuclei with dispersed chromatin, as well as abundant pale or eosinophilic cytoplasm. Emperipolesis with small lymphocytes, plasma cells and, rarely, red blood cells in the cytoplasm of the histiocytes (40×). **(d)** Hassall’s corpuscles identified in the tissue

**Fig. 3:**
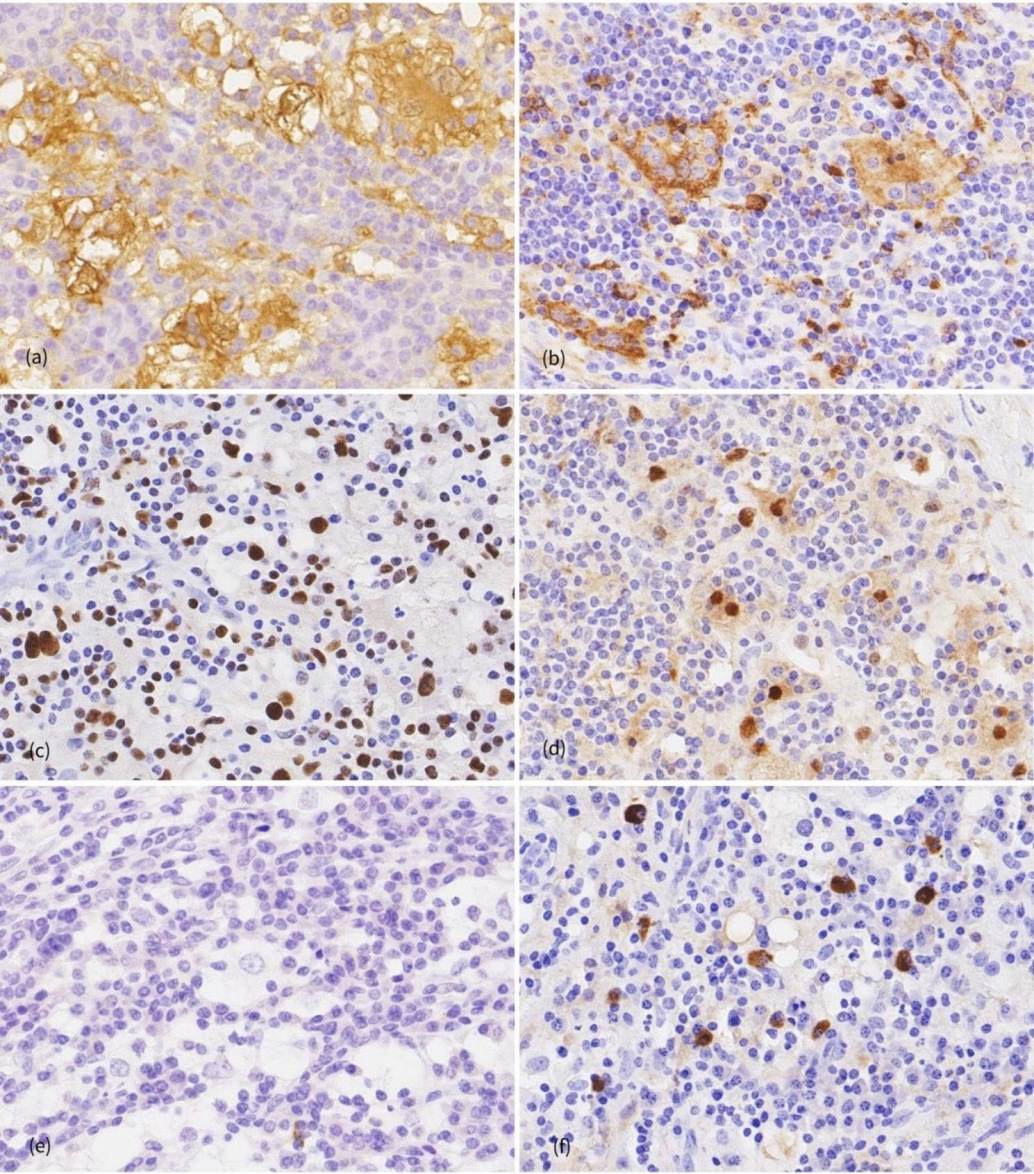
Immunohistochemical features of primary thymic RDD. The large histiocytes were positive for S100 **(a)**, CD163 **(b)**, OCT2 **(c)** and cyclin D1 **(d)** but negative for CD1a (e; 40×). **(f)** In addition, we identified only scattered positive plasma cells for IgG4 (40×)

Formalin-fixed resection specimens were processed according to a routine protocol, embedded in paraffin, and stained with hematoxylin and eosin. Histologic sections revealed clusters and sheets of histiocytes with abundant cytoplasm, accompanied by lymphocytes and plasma cells with interstitial fibrosis (Fig. 2a,b). The histiocytes exhibited large oval or round nuclei with dispersed chromatin, as well as small prominent nucleoli with few mitotic figures. The histiocytes displayed abundant pale or eosinophilic cytoplasm, and engulfed small lymphocytes, plasma cells and rarely red blood cells within the cytoplasm—findings consistent with emperipolesis (Fig. 2c,d). Normal thymus tissue was identified in the lesion (Fig. 2d). Immunohistochemical staining revealed that the histiocytes were positive for S100, CD68, CD163, OCT2 and cyclin D1, but negative for both CD1a and BrafV600E (Fig. 3a-e). In addition, we identified only scattered positive plasma cells for immunoglobulin G4 (IgG4), which showed a low IgG4/IgG ratio (Fig. 3f). The patient was discharged 3 days after the surgery with no complications. In a regular follow-up examination 1 year after the surgery, no evidence of recurrence was detected.

## Discussion

RDD is currently considered a neoplastic disease with clonal proliferation of histiocytes in the lymph nodes or extranodal tissue with unknown cause. RDD can occur in people of any age but is diagnosed most frequently in children and young adults (mean age, 20.6 years) and shows a slight male predominance [5]. RDD is diagnosed more frequently in Black and white than Asian patients [2]. Although RDD was initially described as a painless adenopathy of the cervical and inguinal lymph nodes, 25–40% of RDD cases present with only extranodal manifestations [2, 6, 7]. RDD presenting as an isolated lesion in the thymus is a very rare extranodal form of this disorder; to date, approximately nine such patients have been reported in the English language literature (Table 1) [8–16]. The mean age of these nine patients was 44 years (range, 23–70 years). Two patients presented with typical clinical features of RDD, including a neck mass and fever. All nine patients underwent surgical excision of the primary lesion. At the last follow-up, five of the patients remained alive and showed no further evidence of recurrence of this disorder, whereas one patient showed deterioration secondary to a presumably unrelated malignant tumor (Table 1).

**Table 1:** Clinicopathological features of primary thymic Rosai-Dorfman disease (RDD)

Reference	Age (y)/ sex	Clinical presentation	Lesion size	Therapy	Outcome
Lim et al., 2004 [8]	43/M	Left neck mass	2.0 cm	Resection	n.a.
Wu et al., 2011 [9]	42/M	Fever, fatigue, poor appetite, splenomegaly and thrombocytopenia	n.a.	Resection	Deteriorated secondary to diffuse large B-cell lymphoma in the spleen
Raslan et al., 2011 [10]	50/F	Galactorrhea, headaches	n.a.	Resection	n.a.
Wang et al., 2015 [11]	30/M	Asymptomatic	2.0 × 1.5 × 1.0 cm	Resection	Alive
Tsujimura et al., 2021 [12]	70/F	Asymptomatic	2.7 × 1.5 × 1.2 cm	Resection	Alive
Shen et al., 2021 [13]	49/F	Asymptomatic	1.7 × 1.5 cm	Resection	Alive
Jia et al., 2022 [14]	23/M	Chest pain	8.5 × 5.0 cm	Resection	n.a.
Oramas et al., 2022 [15]	42–47/M	Cough, mild dyspnea and chest pain	4.0 cm	Resection	Alive
Liu et al., 2023 [16]	48/F	Asymptomatic	3.0 × 2.2 cm	Resection	Alive
Present case	64/M	Asymptomatic	1.2 × 1.0 cm	Resection	Alive

Abbreviations: n.a., not available

RDD occasionally manifests as a multifocal and persistent disease with systemic symptoms including fever and weight loss [2, 17, 18]. RDD can also develop in patients diagnosed with other immunological disorders, including systemic lupus erythematosus, idiopathic juvenile arthralgias and autoimmune hemolytic anemia [19]. The results of laboratory tests and radiographic studies are generally unremarkable, although increased ESR, polyclonal gammopathy and neutrophilic leukocytosis have been observed [2, 18]. None of these findings were present in our patient.

RDD in the lymph nodes is characterized in sinusoidal infiltration by S100-positive and CD68/CD163-positive histiocytes. Emperipolesis, “the active penetration of one cell by another which remains intact,” is a classical feature of RDD in the lymph nodes but is less frequently detected at extranodal sites [20, 21]. Because primary thymic RDD is very rare, clinicians and radiologists usually have difficulty in making a definitive diagnosis without pathologic confirmation. Histopathologic features of RDD are usually pathognomonic, including histiocytic proliferation in the sinus of lymph nodes or extranodal soft tissue. The distinctive large histiocytes exhibit abundant cytoplasm, with engulfed small lymphocytes, plasma cells and, rarely, red blood cells. An immunostaining panel is usually used to confirm the diagnosis, including S100, CD68, CD163 and CD1a [21]. As we noted, the expression of CD163 is stronger than CD68 in RDD. Recently, OCT2, a B-cell and monocyte transcription factor that undergoes downregulation after differentiation of monocytes and dendritic cells, has been confirmed to be overexpressed in RDD [4, 22]. Use of antibodies against OCT2 is currently recommended in the immunostaining panel for the diagnosis of RDD. Furthermore, recent studies have identified mutations of MAPK pathway genes in RDD, including *KRAS*, *NRAS*, *HRAS*, *ARAF*, *BRAF* and *MAP2K1* [19, 21, 23]. Cyclin D1 is a biomarker associated with activating mutations of genes in the MAPK pathway and phosphorylated extracellular signal-regulated kinase (pERK) gene mutation. Cyclin D1 overexpression has been identified in RDD. However, recent studies have reported only variable detection of pERK. This finding suggests that all RDD cases might not arise from gene mutations in the MAPK pathway. Recent studies have suggested that overexpression of cyclin D1 may be associated with loss of exon 5 in the CDC73 gene, and may play a role in the pathogenesis of RDD [4, 24].

Langerhans cell histiocytosis should be considered a major differential diagnosis [25]. That disorder can be distinguished from RDD through its characteristic cytologic feature of Langerhans cells and consistent expression of CD1a, Langerin and ZBTB46; however, most cases do not express either OCT2 or CD163 [4]. Likewise, RDD can be quite difficult to distinguish from extranodal cases of Erdheim-Chester disease (ECD), another histocytic disorder with clonal proliferation of lipid-laden macrophages. BrafV600E mutation is detected in most ECD cases. Clinical presentation varies, and multiple organs may be involved, including bone, lungs, brain and skin, with bone sclerosis, pulmonary fibrosis and symptoms associated with central nervous system involvement [21]. The histiocytes in ECD show foamy cytoplasm and express CD14 and Factor 13a, but not S100 and OCT2 (Table 2) [4].

**Table 2:** Immunophenotypic features of diseases in the differential diagnosis of RDD.

	S100	CD 163	CD 68	CD1a/ Langerin	ZBT B46	OCT2	Cyclin D1		Factor 13a	CD 30
RDD	+	+/-	+			+		+	-/+	-/+
LCH	+	-/+	+	+	+	-/+		+		
ECD	-/+	+	+					+	+	

+/-: biomarker positive in most cases

-/+: biomarker positive in few cases

Abbreviations: RDD, Rosai-Dorfman disease; LCH, Langerhans cell histiocytosis; ECD, Erdheim-Chester disease

Variable numbers of lymphocytes, plasma cells, neutrophils and even eosinophils may accumulate in RDD lesions. CD30 expression is observed in the histiocytes in as many as half of all cases. In several cases, Hodgkin lymphoma (HL) should be another differential diagnosis for RDD. CD30-positive histiocytes in RDD show cytoplasmic staining, whereas Hodgkin cells usually show characteristic cell membrane staining with paranuclear Golgi staining [5]. Furthermore, the expression of OCT2 and other B-cell markers can be used to distinguish HL from RDD.

An increase in plasma cells, particularly immunoglobulin G4 (IgG4)-positive plasma cells can be detected in the tissue of RDD. Therefore, IgG4-related disease may be considered in the differential diagnosis. IgG4-related disease usually shows tissue infiltration by mixed small lymphocytes and plasma cells with storiform fibrosis and obliterative phlebitis. Elevated serum IgG4 levels may confirm the diagnosis. The plasma cells in both RDD and IgG4 related disease are polyclonal. No clear evidence suggests that IgG4-related disorders and RDD share the same pathogenesis [21, 23, 26].

The clinical course of RDD is usually indolent and self-limited; the symptoms may gradually subside over months to years, or in response to therapy. Occasionally, patients with RDD might show recessive or refractory courses, which can rarely be fatal. Although cases of RDD associated with lymphoma (e.g., HL and follicular lymphoma) have been reported, the literature has not documented increased risk of lymphoma in RDD [6]. Consensus recommendations for the management of RDD were published in 2018. Current recommendations suggest tailored treatment according to individual patient and clinical circumstances. Observation is suitable in cases without complicated nodal and cutaneous disease, because most of these patients will undergo spontaneous remission. Surgical resection is curable for most patients with unifocal extranodal disease. Surgical resection in some patients can decrease symptoms and improve the function of organs affected by RDD. Several postoperative regimens have recently been evaluated and may be beneficial for patients, including the use of corticosteroids, sirolimus, chemotherapy, radiotherapy, or immunomodulatory and targeted therapies [21].

## Conclusion

In summary, we report a rare case of unifocal extranodal RDD occurring in the thymus. Thymic RDD appears no different from RDD at other anatomic sites, in terms of histolopathologic features and clinical course. Additional case studies are needed to determine whether thymic RDD might be a unique subtype of the disease.

## Data Availability

Not applicable.

## Abbreviations

RDD: Rosai-Dorfman disease
MAPK: Mitogen-activated protein kinase
pERK: Phosphorylated extracellular signal-regulated kinase
ECD: Erdheim-Chester disease
HL: Hodgkin lymphoma
IgG4: Immunoglobulin G4
